# Violence, mental health and violence risk factors among women in the general population: an0020epidemiology study based on two national household surveys in the UK

**DOI:** 10.1186/1471-2458-13-1020

**Published:** 2013-10-29

**Authors:** Min Yang, Stephen CP Wong, Jeremy W Coid

**Affiliations:** 1 School of Public Health, Huaxi Medical Centre, Sichuan University, No 17, Section 3, South Renmin Road, Chengdu, China; 2 Institute of Mental Health, University of Nottingham, The Innovation Park of Nottingham, NG7 2TU Nottingham, UK, England; 3 Forensic Psychiatry Research Unit, Queen Mary’s University London, William Harvey House, 61 Bartholomew Close, EC1A 7BE, London, UK, England

**Keywords:** Female violence, Mental disorders, Risk factors, Community samples, UK

## Abstract

**Background:**

Females who perpetrated violence in the community have important mental health and public protection implications. There is a dearth of research in this area. This study investigated the prevalence of psychiatric morbidity, personality disorders as well as victim characteristics and violence risk factors of women in the community who self-reported violence against others.

**Methods:**

The study sample consisted of 8,275 community women aged 16–74 years obtained from the 2000 and 2007 UK national psychiatric morbidity surveys. Self report incidences of violence, personality disorders and the presence of psychiatric symptoms were assessed by interviews and/or established structured psychiatric assessment protocols.

**Results:**

Weighted prevalence of female violence, which primarily involved partners and friends, was 5.5% in 2000 and 5.1% in 2007. Violence-prone women also had significantly higher prevalence of common mental disorders and comorbidity (adjusted odds ratio 3.3 and 2.9 respectively) than non-violent women. Multivariate analyses identified eight significant risk factors that characterized violence prone women: young age, residing in social-assisted housing, presence of early conduct problems, victim of domestic violence, self-harming, excessive drinking and past criminal justice involvements.

**Conclusion:**

A higher prevalence of common mental disorders and some types of personality disorder was found among violence prone women compared to their non-violence prone counterparts. The identified violence risk factors could be used to develop a quick and easily administered rating tool suitable for use by non-mental health trained frontline workers such as police and social support workers in the community to identify violence-prone women. Mental health and support services then can be provided to them for mental health care and violence prevention purposes.

## Background

Violence prevention has important implications for public safety, health and criminal justice services. The WHO recommended that the health and criminal justice sectors should take a more proactive role in violence prevention [1]. An effective public health approach should include the development of a comprehensive understanding of the magnitude, scope, characteristics and consequences of the many facets of violence based on empirical research, the results of which then can be used in violence prevention and intervention. Within the criminal justice sector, studies of violence and violence prediction have predominantly focused on violence perpetrated by high risk, male offenders or those with forensic mental health problems. Results from these studies have led to the development of violence prediction tools and effective intervention programmes [2] to reduce violence among male offenders and patients. However, there has been debate over whether risk predictors developed for male offenders can be generalized and applied to women [3]. A limited number of studies have identified risk factors for female violence but the studies were based on incarcerated women [4,5]. Research on assessing risks of violence in non-incarcerated women is very limited [6].

In the public health sector, violence perpetrated by women living in the community is not well understood because of the lack of research. This is, in part, because women in the community are difficult to access and also because women are predominantly portrayed as victims rather than perpetrators of violence [7]. A search of the literature between 1990 to 2011 using key words 'female violence’ or 'female perpetrators’ or 'women violence’ or 'women perpetrators’ returned only sixteen papers, among which only four were based on community samples. Of the four, two were large scale cross-sectional surveys [8,9] and two comprised of small college student samples [10,11]. All four studies with community samples focused on different aspects of intimate partner or domestic violence. There were few large cross-sectional nationwide surveys that examined the prevalence of violent acts among both men and women in the community using an epidemiological approach [11-14]. One UK study examined mental health and psychosocial risk factors for both men and women that allows for a comparison between the sexes [15]. As expected, men have a higher incidence of self reported violence against others (15%) than women (5.5%). This study also found that, compared to men, women who self-reported violent acts demonstrated more symptoms for affective and anxiety disorders and personality disorder, but fewer symptoms for alcohol dependence and hazardous drinking.

Despite rapid social changes, women in both developed and developing countries continue to assume the role of primary carer of children irrespective of income level. As such, violence prone women in the community are potentially at risk for endangering the children under their care as evidenced by a number of tragic and high profile cases that involved the serious abuse of children by their mothers despite interventions by social care and law enforecement agencies e.g. [16]. Needless to say, the safety and welfare of children are paramount in any society. An epidemiological study of violence-prone women in the community can provide information on the prevalence of the problem, the characteristics of at-risk women and their victims, and the situational context of their violent acts. With further validation studies, the identified risk factors can be used in the early detection of women at risk such that they can be provided with support and assistance to reduce the potential harm to themselves and others, in particular, to children under their care.

Because of the low prevalence of women perpetrators of violence in the community, only epidemiological studies with sufficiently large population samples can provide reliable evidence to answer the necessary research questions discussed above. The present study aims to achieve the following objectives based on data from two large, nationally representative samples of adult women selected from the general household population in the UK. First, we report the prevalence of female violence in the community; second, we compare differences in psychiatric morbidity between violent and non-violent women; and, third, we identify and cross validate risk factors for female violence in the community with the aim of developing a risk prediction tool that can be used by front line health and support services providers to identify at risk women.

## Methods

### Sample

The study samples were taken from the 2000 and the 2007 National Household Surveys of Adult Psychiatric Morbidity in the UK^a^. The 2000 survey consisted of 4,634 women respondents aged 16–74 and the 2007 survey, 3,641 women in the same age range. The 2000 survey was undertaken in England, Scotland and Wales by the Office for National Statistics (ONS) and was commissioned jointly by the Department of Health, Scottish Executive and National Assembly for Wales [17]. The 2007 survey collected data from adults in England only and was carried out by the National Centre for Social Research in collaboration with the University of Leicester and was commissioned by the National Health Service Information Centre for Health and Social Care [18].

Both surveys were originally designed to assess, among adults living in private households, the prevalence of psychiatric morbidity as well as the changes in psychiatric morbidity and the use of health services over time. To compare changes in psychiatric morbidity over time, both surveys used the same sampling frame unless indicated, the same inclusion and exclusion sampling criteria to select individuals, and the same measurements for psychiatric disorders, including substance use disorders. Persons incarcerated or living in institutions were excluded from both surveys. Both surveys used computer assisted interview in two phases with small-user Post Address Files as the sampling frame and Kish method to select one person systematically in each household. An individual weighting factor was derived to take into account the proportions of non respondents according to age, sex, and region to ensure a sample representative of the national population and compensating for sampling design. Details of sampling and survey methods for both surveys can be found elsewhere [17,18].

### Measurement of violent behaviour

All subjects were asked questions about the presence of violent behaviour in the context of establishing the diagnosis of antisocial personality disorder (ASPD; *Diagnostic and Statistical Manual of Mental Disorder*, Fourth Edition (DSM-IV; 1994) using the Structured Clinical Interview Axis II Disorders (SCID-II) screening for ASPD. In addition to the ASPD criteria, subjects were also asked the question: "*Have you been in a physical fight, assaulted or deliberately hit anyone in the past five years*?" An affirmative answer was followed by further questions on the total number and location of incidents, the number of victims, the relationship of the perpetrator to the victims and any victim injury. These questions were used in previous surveys in New York [13] and Israel [12]. Any woman who gave a positive answer to the lead question was deemed to be violence-prone and allocated to the violent category.

### Measurement of common mental disorders (CMDs)

Common mental disorders included generalized anxiety disorder, mixed anxiety and depressive disorder, all phobias, obsessive-compulsive disorder, panic disorder and depressive episode. The revised version of the Clinical Interview Schedule (CIS-R) [19] was used to assess if these conditions, which are not mutually exclusive, were present in the past week. The CIS-R was administrated using Computer Assisted Personal Interviewing in the 2000 and 2007 surveys. The probably presence of psychosis was assessed using the Psychosis Screening Questionnaire in both surveys.

### Measurement of substance misuse disorders

The Alcohol Use Disorder Identification Test (AUDIT) was used to assess three primary categories of alcohol disorder in the past year: hazardous alcohol use, harmful alcohol use, and alcohol dependence. The AUDIT questionnaire was administrated using computer-assisted self-completion interview (CASI). Questions about alcohol consumption were scored from zero to 40. AUDIT score of 8 or more indicates hazardous drinking problems. For those with AUDIT scores of 10 or more, the Severity of Alcohol Dependence Questionnaire (SADQ) was included to measure alcohol dependence at 4 levels: none, mild, moderate and severe, over the past year in the 2000 survey. The community version of SADQ or SADQ-C measured alcohol dependence in the last 6 months in the 2007 survey. Any alcohol dependence was defined in this study based on the SADQ designated levels of mild, moderate, or severe.

Questions on lifetime drug use and drug use in the past year were asked using CASI in both surveys. Positive responses to questions on the dependence on different substances (including cannabis, amphetamines, cocaine, crack cocaine, ecstasy, tranquilisers, opiates, and volatile substances) were combined to produce a single category of "any" drug dependence. The same measure was used in both surveys.

### Risk factors for violence prone women

Previous research with women offenders indicate risk predictors can be classified into six domains: *pathway aetiology* including childhood adversity and conduct problems; *women’s social living and relationships*; *victimisation history*; *antisocial life style*; *adult trauma and mental health problems*; and *substance abuse*[20]. Empirical studies have yet to demonstrate that these risk prediction domains which are derived from studies of incarcerated women can generalize to women in the community. Guided by the constructs within these six domains, 52 variables^b^ that are easy to obtain from the respondents themselves or their family members or friends and best mapped onto the six domains were culled from the 2000 survey sample by the authors. The association of significant factors with self-reporting violence was firstly examined using stepwise logistic regression analysis by each category separately. Variables showed significant association with the violence outcome from each category were then pooled together in one stepwise logistic regression model to establish their independent effects on the outcome.

### Statistical analysis

For overall prevalence of female violence and psychiatric morbidity, case weighting was used in the computation to reflect the proportional sampling procedure. Technical details for the weighting procedure are published elsewhere [17,18]. Chi-squared test was used to test difference in proportions, such as rates of unemployment, type of violence victims between 2000 and 2007 surveys. Odds-ratio (OR) was used to measure the association of a psychiatric disorder with violent outcome. Further logistic regression analysis was used to estimate adjusted OR (AOR) of each psychiatric disorder by taking into account confounding or covariates when necessary.

Identification of variables that best predicted violence-prone women was based on the sample of 4,634 women in the 2000 survey. Univariate analysis of 52 variables was conducted first to screen in fewer variables significantly associated with the violence outcome. For variables with their OR greater than 2 and significant level p < 0.05 in the univariate analysis, backwards stepwise logistic regression analysis of them was used to develop a final model with only variables that were independently predictive. The predictive effect of the final model was assessed by the area under the curve (AUC) value derived from receiver operating characteristic curve analysis. The goodness of fit of the model was tested by the Hosmer & Lemeshow calibration approach [21]. The external predictive accuracy of these factors was then revalidated on the sample of 3,641 women in the 2007 survey.

All analyses were performed in SPSS v17.0 for Window XP.

## Results

### Prevalence of female violence and demographics of violent prone women

**Figure 1:**
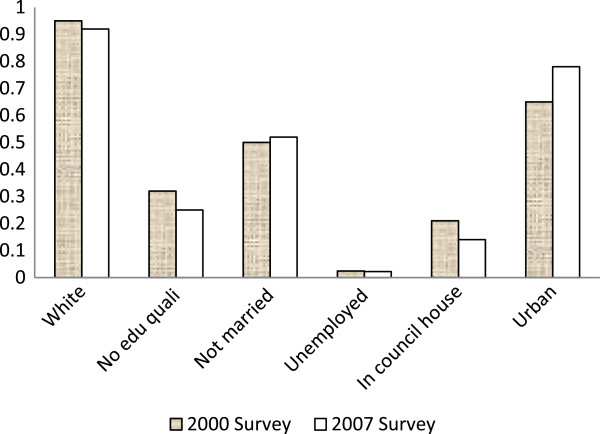
Distribution of women in percentage by demographics in the two surveys.

The mean age of the 4,634 female respondents in the 2000 sample was 45.4 years (SD = 15.6) and that of 3,641 females in the 2007 sample was 46.2 (SD = 15.3). In the 2000 and 2007 surveys, 246 and 158 women reported violent incidents, respectively. The weighted prevalence of violence was 5.5% (95% C.I.: 4.8 – 6.2%) and 5.1% (95% C.I.: .4.4 – 5.8%) respectively with no difference in prevalence (χ^2^ = 0.798, p = 0.37) between the two surveys. Because the 2007 survey covered only households in England, we calculated prevalence of female violence for England and for Scotland and Wales separately for the 2000 survey. There was no difference between the regions, with a prevalence of 5.3% for England and 5.5% for Scotland and Wales (Chi-squared = 0.09, p = 0.76). The two samples have similar demographic characteristics (Figure 1).

Compared to women with no self-reported violence (Table 1), violence-prone women were on average 15 years younger, more likely unmarried, and lived in council (government subsidized) properties in an urban environment. There was no difference in ethnicity and recorded rates of unemployment between the two groups of women. The differences or lack thereof between violence-prone women and non-violent women were very similar in both survey samples, except for educational qualification which were higher among violence-prone women in the 2000 survey. There were no differences in the 2007 survey.

**Table 1:** Demographic differences between violent and non-violent women

	**2000 survey**			**2007 survey**		
	**Violent N = 246**	**Non-violent N = 4,382**	**P value**	**Violent N = 158**	**Non-violent N = 3,427**	**P value**
White: %	94.7	95.0	NS	89.9	92.2	NS
No education qualification: %	24.0	32.2	<0.01	21.5	24.9	NS
Not married: %	81.7	47.9	<0.001	82.9	50.4	<0.001
Unemployed: %	4.1	2.3	NS	4.4	2.0	NS
In council housing: %	42.7	19.8	<0.001	32.9	13.3	<0.001
Urban: %	73.2	64.9	<0.05	82.9	77.7	<0.001
Age: Mean (SD)	30.1 (9.9)	46.3 (15.4)	<0.001	31.4 (11.8)	46.9 (15.1)	<0.001

### Characteristics of victims and violent incidents

Table 2 presents characteristics of victims and violent incidents based on subgroup analysis of violence-prone women only. The variables in Table 2 are not mutually exclusive. An act of violence could involve multiple persons with multiple forms of injury. Results of the two surveys are highly comparable, with the exception of more stranger-victims, fewer victims injured, and fewer participants reporting five or more incidents of violence in 2007 compared to 2000. Taking the two samples together, partners and acquaintances constituted the vast majority of victims of female violence: 81.3% in 2000 and 77.8% in 2007. It was notable that children were reported as victims, but at a much lower rate. The results are self-reported acts of violence and would likely reflect an under-estimation of such occurrences because of society’s strong sanctions on such behaviours.

**Table 2:** Characteristics of victims and violent incidents reported by violent-prone women

	**2000 Survey**	**2007 Survey**	**Sig. P**
	**n (%)**	**n (%)**	
**Type of victim**			
Partner	116 (47.2)	75 (47.5)	0.95
Friends/People known	89 (36.2)	65 (41.1)	0.27
Other family members	34 (13.8)	25 (15.8)	0.58
Children	12 (4.9)	9 (5.7)	0.72
Strangers	59 (24.0)	51 (32.3)	0.07
**Location of violence**			
Home	143 (58.1)	86 (54.4)	0.46
Street/Pub/Bar	138 (56.1)	93 (58.9)	0.58
Work place	8 (3.3)	5 (3.2)	0.96
Other place	20 (8.1)	15 (9.5)	0.63
**Injury**			
Participant injured	69 (28.0)	55 (34.8)	0.15
Victim injured	42 (17.1)	13 (8.2)	0.01
**Repetition**			
Three or more victim types	10 (4.1)	9 (5.7)	0.45
Five or more incidents	35 (14.2)	7 (4.4)	0.002
**Violent – prone women (Base number)**	246	158	

Results from Table 2 show that the locations of violence were about evenly divided between the home (56.7%) and street/pubs (57.2%). Assaulting partners was somewhat higher in the home (47.3%) than in street/pubs (34.1%), with the reverse observed for general assaulting (51.4% street/pubs vs 38.1% home). The workplace was the least likely location for female violence. Distributions of violence by location were similar for 2000 and 2007. With regard to injury suffered, more women perpetrators were themselves injured (30.7%) than their victims (13.6%) in violent incidences reported in both surveys. Thirty eight women out of 69 (55.1%) who suffered from injuries did so primarily through the actions of their partners and 25 (36.2%) of them through the actions of their friends in the 2000 survey, which was similar to the 2007 survey, with 41.8% and 40.0% respectively. The prevalence of injury inflicted by strangers was significantly higher in the 2007 than 2000 survey (χ^2^ = 7.85, p < 0.01). Among injured victims of the women who reported violence, distributions of victim type showed no statistical difference between the two surveys, with 28.6% and 46.2% reporting injury to partner in 2000 and 2007 respectively (χ^2^ = 1.39, p > 0.10), 61.9% and 53.8% to friends (χ^2^ = 0.27, p > 0.10), and 31.0% and 46.2% to strangers (χ^2^ = 1.01, p > 0.10).

### Psychiatric morbidity

Compared to non-violent women, violence-prone women reported significantly higher prevalence of all psychiatric morbidity in both surveys as shown in the first two columns in Table 3. The differences of the two groups, assessed by the odds-ratio (OR) measure, was presented in the third column in the table. As non-violent women were treated as the baseline, an OR significantly greater than 1 suggests that violent-prone women are more likely to suffer from the psychiatric condition than non-violent women. In the 2000 sample, violent-prone women were more likely to suffer from all psychiatric conditions than non-violent women, and such effects remained significant after taking into account demographic differences and other potential confounds using the adjusted odds-ratio (AOR). The same pattern was found in the 2007 sample, except for probable psychosis, panic and obsessive compulsive disorders, in which the AOR did not show statistical difference. This could be attributed to the low prevalence of these conditions and a smaller sample of the 2007 survey. Similar patterns between the two samples were found in the diagnosis of drug dependence (AOR: 3.2 in 2000 and 3.8 in 2007), phobias (AOR 2.4 and 3.7 respectively), depressive episode (AOR 3.7 and 2.0 respectively), and neurotic disorder (AOR 1.8 and 2.1 respectively), suggesting that overall violent-prone women were more likely to suffer from those conditions than non-violent women. The risk of alcohol dependence among violence-prone women was estimated to be greater in the 2000 sample (AOR 4.8) than in the 2007 sample (AOR 1.8). This could be due to the different reference periods used to measure the condition – (past 6 months in 2007 versus past 12 months in 2000). Similarly, the difference between the two samples in the risk of any personality disorder (PD) was most likely due to the fact that the 2000 survey used SCID II screen for all Axis II PDs whilst the 2007 survey only included diagnoses of ASPD and Borderline PD.

**Table 3:** Weighted prevalence of psychiatric morbidity among violent and non-violent women

**Conditions**	**Violent%**	**Non-violent%**	**OR (95% CI)**	**AOR (95% CI)**^**a**^
**2000 survey**	**Weighted**	**Weighted**		
	**N = 233**	**N = 3,984**		
Probable Psychosis	1.7	0.4	4.6 (1.5 – 14.0)	6.2 (1.9 – 20.4)
Mixed anxiety and depression	18.0	10.3	1.9 (1.4 – 2.7)	1.6 (1.1 – 2.4)
General anxiety	7.7	4.3	1.9 (1.1 – 3.1)	2.6 (1.5 – 4.6)
Panic	1.7	0.7	2.7 (0.9 – 7.7)	3.5 (1.1 – 11.1)
Obsessive compulsive	3.9	1.1	3.5 (1.7 – 7.3)	2.8 (1.3 – 6.3)
All phobias	4.7	2.0	2.5 (1.3 – 4.7)	2.4 (1.2 – 4.7)
Depressive episode	8.6	2.3	4.1 (2.5 – 6.7)	3.7 (2.1 – 6.5)
Drug dependence	12.0	1.6	8.4 (5.3 – 13.5)	3.2 (1.9 – 5.4)
Alcohol dependence	15.1	2.2	7.9 (5.2 – 11.9)	4.8 (3.0 – 7.6)
Adult antisocial PD	11.2	1.7	7.2 (4.5 – 11.6)	3.8 (2.2 – 6.6)^b^
Screen + v for any PD	49.8	25.7	2.8 (2.2 – 3.7)	2.2 (1.6 – 3.0)^b^
Any neurotic	35.6	18.2	2.5 (1.9 – 3.3)	1.8 (1.3 – 2.5)^c^
**2007 survey**	**Weighted**	**Weighted**		
	**N = 173**	**N = 3,223**		
Probable Psychosis	1.2	0.6	2.7 (0.8 – 9.2)	2.3 (0.6 – 8.2)
Mixed anxiety and depression	24.9	10.8	2.8 (1.9 – 4.0)	2.4 (1.7 – 3.6)
General anxiety	10.9	5.3	2.1 (1.3 – 3.6)	1.9 (1.1 – 3.2)
Panic	1.1	1.3	0.7 (0.1 – 3.4)	0.6 (0.1 – 2.8)
Obsessive compulsive	3.5	1.2	2.9 (1.2 – 6.8)	1.5 (0.6 – 3.6)
All phobias	8.6	1.9	4.8 (2.6 – 8.7)	3.7 (1.9 – 7.1)
Depressive episode	4.6	2.7	1.7 (0.8 – 3.6)	2.0 (1.0 – 3.8)
Drug dependence	12.7	2.0	7.4 (4.4 – 12.3)	3.8 (2.2 – 6.6)
Alcohol dependence	9.8	3.3	3.2 (1.8 – 5.5)	1.8 (1.0 – 3.9)
Adult antisocial PD	17.3	6.7	2.8 (1.9 – 4.3)	1.8 (1.2 – 2.9)^b^
Screen for ASPD or BPD^d^	22.0	2.4	11.1 (7.2– 16.9)	4.3 (2.7 – 3.8)^b^
Any neurotic	43.9	19.3	3.3 (2.4 – 4.5)	2.1 (1.5 – 3.1)^c^

^a^Adjusted for age, marital status, accommodation and area; ^b^Adjusted for age, marital status, accommodation, area lived in and neurotic disorder; ^c^Adjusted for age, marital status, accommodation, area lived in and any PD; ^d^Only two PDs, ASPD and borderline PD were available in the 2007 survey.

To further assess the association between co-morbidity of CMD and female violence, all participants were further grouped into four mutually exclusive categories: (A) no CMD condition, (B) any single condition, (C) any two conditions, and (D) 3–6 conditions. Results in Table 4 demonstrate a significantly increased prevalence of female violence as the number of CMD diagnostic co-morbidity increased, in both surveys. This ranged from 4.4% for women without CMD condition to 16.6% for women with 3 or more conditions in the 2000 survey (χ^2^ = 53.5, p < 0.0001 for linearity test), and from 3.6% to 12.5% (χ^2^ = 48.4, p < 0.0001 for linearity test) in the 2007 survey. In the 2000 sample, AORs for the risk of self-reported violence, after adjusting for demographic differences, also increased from 2.1 to 5.1 as the CMD co-morbidity increased. When analysing associations of any CMD condition (none or any) with self-reported violence, we observed OR 4.4 (95% CI 3.3 – 5.7) for the 2000 sample and 3.9 (95% CI 2.8 – 5.4) for the 2007 sample. The AOR, adjusted for demographics, was 3.3 (2.5 – 4.4) and 2.9 (2.1 – 4.2) for the two samples respectively.

**Table 4:** Weighted prevalence and co-morbidity of common mental disorders and associations with violence

	**2000 survey**			**2007 survey**		
**Number of CMD**	**All women**	**Violence prone women, n(%)**	**AOR (95% CI)**^**a**^	**All women**	**Violence prone women, n(%)**	**AOR (95% CI)**^**a**^
0	3409	150 (4.3)	1.0 (Ref)	2700	97 (3.6)	1.0 (Ref)
1	721	68 (9.4)	2.1 (1.5 – 2.8)	591	66 (11.2)	2.8 (2.0 – 4.0)
2	65	12 (18.5)	6.0 (2.9 – 12.3)	73	6 (8.9)	2.1 (0.9 – 5.2)
3 – 6	24	4 (16.6)	5.1 (1.5 – 16.8)	32	4 (12.5)	2.4 (0.7 – 7.9)

^a^Adjusted for age, married or not, education level, type of accommodation, social class and IQ score.

### Risk factors for violence prone women

Univariate analysis of the 52 variables identified 16 that were significantly associated with violence prone women, with odds ratios (OR) between 2.4 to 15.6, as shown in Table 5 by six domains.

**Table 5:** Risk factors of female self-reported violence - univariate analysis (2000 survey)

**Risk factors**	**Violent (Base: 246) N(%)**	**Non violent (Base: 4,388) N(%)**	**Odds-Ratio (95% C.I.)**
**Childhood adversity and conduct acts**			
Ran away	63 (25.6)	200 (4.6)	7.21 (5.24–9.92)
Institutional care before age of 15	23 (9.3)	123 (2.8)	3.58 (2.25–5.69)
School expels	44 (17.9)	265 (6.0)	3.39 (2.39–4.80)
Conduct problems before the age of 15^a^	52 (21.7)	76 (1.7)	15.6 (10.7–22.9)
**Demographics**			
Ever had a paid job	14 (13.7)	121 (6.3)	2.36 (1.30–4.26)
Unstable relationship or unmarried	201 (81.7)	2104 (47.9)	4.85 (3.50–6.76)
Young age (<30 years)	138 (56.1)	762 (17.4)	6.08 (4.67–7.92)
Living in Council housing or ever home less	116 (47.2)	955 (21.8)	3.21 (2.47–4.16)
**Victimisation**			
Ever experienced sex abuse	38 (15.4)	237 (5.4)	3.20 (2.21–4.63)
Ever experienced domestic violence	95 (38.6)	435 (9.9)	5.72 (4.34–7.53)
**Adult Antisocial (AS) life style**			
Had problems with Police	39 (15.9)	125 (2.8)	6.43 (4.37–9.45)
AS lifestyle^b^	24 (9.8)	71 (1.6)	6.57 (4.06–10.7)
**Mental/Emotional problems/Trauma**			
Ever attempted self-harm/suicide	65 (26.4)	250 (5.7)	5.94 (4.36–8.11)
Traumatised from separation/devoice	137 (55.7)	1421 (32.4)	2.62 (2.03–3.40)
**Substance use problems**			
Any drug dependence	29 (11.8)	60 (1.4)	9.62 (6.05–15.3)
Hazardous drinking	97 (39.6)	585 (13.4)	4.25 (3.24–5.57)

^a^Presence of any three of the followings: bully others, fights, used weapon, canning, tortured others, stealing, mugging, breaking in, vandalism, fire setting, cruel to animals, forced sexual activity.

^b^Presence of any three of the followings: stealing or selling drugs or having sex for money or lied to get what you want or drunk driving or no remorse for wrong doing.

Further backwards stepwise logistic regression analysis of the 16 risk factors yielded a final set of 8 variables significantly associated with violent outcomes (Table 6). These variables covered all six domains with 2 variables each for demographic and mental/emotional problems/trauma, and one for each of the remaining four domains as shown in Table 5. The strength of association of these risk factors with self-reported violence can be classified in three groups. In descending order of magnitude, the groups are: young age and conduct problems before 15 in the first group, followed by victim of domestic violence, problems with police due to criminal behaviour, and drinking problems in the second group. The last group included poor accommodation, attempted self-harm or suicide, and traumatisation from broken relationships.

**Table 6:** Risk factors from multivariate logistic regression analysis (2000 Survey)

**Risk factors**	**Coefficient (SE)**	**Odds-Ratio (95% C.I.)**
V1: Conduct problems before the age of 15	1.519 (0.23)	4.57 (2.91–7.18)
V2: Young age (<30 years)	1.645 (0.15)	5.18 (3.84–7.00)
V3: Living in social housing or ever homeless	0.570 (0.16)	1.77 (1.30–2.41)
V4: Ever experienced domestic violence	0.970 (0.18)	2.64 (1.85–3.76)
V5: Had problems with Police	1.009 (0.25)	2.74 (1.70–4.44)
V6: Ever attempted self-harm/suicide	0.560 (0.20)	1.75 (1.18–2.60)
V7: Traumatised by separation/devoice	0.561 (0.16)	1.75 (1.27–2.41)
V8: Hazardous drinking	0.836 (0.16)	2.31 (1.69–3.16)
**Intercept**	-4.596 (0.16)	

All variables are dichotomous and coded 0 for absence and 1 for presence.

A risk score for each woman was calculated based on the model coefficients presented in Table 6. The model estimated probability of committing a violent act was compared with the observed outcome of reporting violence. The predictive efficacy of the risk score measured by AUC value was 0.85 (95% CI: 0.82 to 0.87), indicating a high level of accuracy of the model in discriminating between violent and non-violent women, with a sensitivity of 77.4% and specificity 75.7% at a cut-off -3 of the estimated risk score. The Hosmer – Lemeshow calibration test of 7 individual risk groups was acceptable (χ^2^_6_ = 6.94, p = 0.225).

To further test whether the risk predict model was still valid when used to predict new cases, the risk score based on the same 8 risk factors was also calculated for each of 3,641 women in the 2007 sample. Using the same cut-off as in the 2000 sample, a woman with a score greater than -3 would be predicted as violence-prone. The risk prediction model correctly identified 146 out of 158 (92.4%) women who actually reported violent acts in the 2007 survey, with a false positive rate of 34.1%, AUC value 0.84 (95% CI: 0.81 o 0.86), and H-L calibration statistic 3.07 (p = 0.80). This analysis demonstrated good predictive power of these risk factors when applied to the new (2007) community sample.

## Discussion

To our knowledge, this is the first study that has examined prevalence and patterns of female violence, together with social adversity and mental health problems of violence-prone women, to identify risk factors for female violence using two representative community samples. The findings may fill the gap in our knowledge in the magnitude, context and associates of female violence, and deepen our understanding of female violence in the community. The findings should also provide evidence and guideline for frontline professionals in identifying violent prone women residing in the community in order to help them with social, emotional and medical supports and to prevent them from potential harming their partners, children and friends as well as themselves.

The two surveys, seven years apart, identified a consistent, small but not insignificant number of women in the community (5- 6%) who reported violent behaviour towards others in the past 5 years. Differences between the two surveys in demographics, victim characteristics and psychiatric morbidity of violence-prone women were similar, suggesting consistency in the characteristics of violence-prone women over time and within different regions of the UK.

Before discussing specific findings, several limitations of the study should be considered when interpreting these findings. The prevalence of female violence could have been underestimated due to exclusion of persons in institutions or the criminal justice system. It is also possible that refusal to participate is higher among violence-prone than their non-violence prone counterparts. Recall error may also contribute to low prevalence. Furthermore, in an observational cross-sectional survey, any relationship between violent behaviour and risk factors identified is not causal but associational. In addition, the survey did not have data to differentiate women who acted violently in self-defence in domestic violence from those who were perpetrators and initiated violence.

### Victims of female violence

The majority of victims of violence-prone women were people they knew well, particularly family members and partners. There was a 5% chance of a victim being a child. Of violence towards family members, approximately one-third of violent incidents were directed towards children, the most vulnerable member in the family. These figures, though rather low, are higher than those cited in an official report namely that 1.3% of children under 11 years and 6.9% of youth under 17 years in the UK have self-reported severe physical violence by parents or guardians in their home [22]. The relatively low prevalence of child victim in the present study could be an underestimation given the strong social opprobrium towards such behaviours and should be a significant cause for concern. That such incidents of violence against children were self-reported at all in the surveys is surprising; the results suggest the presence of a significant under-reporting of a child protection problem that requires further investigation. Given there is a strong association between child abuse and domestic violence [23], and given more than half of the violent incidents reported by women in these surveys were domestic violence in nature, there is an additional risk of abuse and violence to children living in such households under the care of violence-prone women.

Our findings generally correspond to studies based on women who report intimate partner violence in the community [9]. As expected, 47% of partners of violence-prone women were victims of violence in both surveys. This suggests a significant overlap in the definition of violence in the present study and intimate partner violence in the literature. We observed that more women than men reported violence against partners in both surveys, although additionally reporting being injured was considerably more common among women who acted violently to their partners than their male counterparts. The instigator of violence is assumed to be the woman on the basis of the survey questions. However, the precipitants and original instigation of aggression was likely complex and may not have been captured accurately by the questions in the survey.

### Violence and mental disorder

Compared to other women, violence-prone women had significantly higher risks of all mental disorders and certain personality disorders. In all, 62.1% of violence-prone women in the 2000 survey and 40.9% in the 2007 survey received at least one diagnosis, and with high levels of co-morbidity. The lower prevalence of psychiatric co-morbidity among the 2007 sample compared to 2000 could be explained by a shorter period for the diagnosis of alcohol dependence and because only two Axis II PD diagnoses (in contrast to 10 categories in 2000) were included in 2007. These findings are largely consistent with studies on women offenders with violent convictions [3,24] and confirm the association between poor mental health and violent behaviour at different levels of seriousness. It is possible that women who reported violent acts in the community and who have mental disorder are at risk of criminal behaviour and/or detection and arrest for such behaviour if there is no intervention for their mental disorder. However, this assumption requires further longitudinal study.

It should be mentioned that the inclusion of only two diagnostic categories of PDs in the 2007 survey was problematic. The prevalence of self-reported violence among women was higher than that of clinical diagnosed ASPD which was in the region of 2 to 3%. The two are not synonymous. Between 11-17% of the violence-prone women were likely to have a diagnosis of ASPD (Table 3). The finding that 50% screened in the 2000 survey with "any" PD, and 22% with either ASPD or BPD in 2007, supports the contention that there are important links between female violence and personality disorders. Unfortunately, we could not explore this further due to difference in categories of PDs included in the two surveys. The relationship between violence and other personality disorders among women requires further investigation.

Although the results do not imply that mental health symptoms were either a direct or indirect cause of violence, the presence of both violence and mental disorder among these women suggests that care and support should be offered to reduce the potential harm to themselves and to others, in particular, to children in their care.

### Prediction and prevention for women at risk for violence in the community

The study identified three groups of risk factors by their magnitude of association with violence behaviour for violence-prone women in the community: historical factors, gender-specific factors, and substance use factors. Among historical/demographic factors, early conduct problems (V1), young age (V2) and contact with police or the criminal justice system (V5) are all well established violence predictors in the risk assessment literature [2] and are often found in commonly-used risk assessment tools for men, such as the HCR-20 [24] and the Violence Risk Scale [25,26]. The presence of these risk factors in the female community sample suggests cross-over of these predictors for men and women.

Experience of domestic violence (V4), attempted self harm/suicide (V6) and traumatisation from broken relationships (V7) in the second group are generally considered female-specific risk factors [2,5]. Alcohol abuse (V8) is well known for its strong associated with violent behaviour for both men and women from research among forensic and non-forensic populations [14,27]. Factors such as V7, V6 and V5 identified in this study are also reported to be significantly associated with severe maltreatment of children and young people by home carers [22]. The risk factors identified in this study suggest that women at risk for violence in the community can be predicted with a moderate to high level of accuracy based on self-report information gleaned from a small number of variables. Based on the risk factors identified, a checklist or similar tool could be developed for the purpose of screening women at risk in community by health professionals or community workers, including social service workers and police without mental health training. Such a tool has a number of merits: firstly, information to rate all the variables can be obtained using self-report and the variables can be easily rated. Second, overall score can be used to trigger referrals to other services. The endorsement of certain variables, such as hazardous drinking, domestic violence, or suicidal attempts, has face validity. These variables suggest potential referral to specific services. Harm from violence could thereby be reduced or averted if frontline workers, including social service workers, GPs, and police can quite accurately identify these women and then refer for appropriate care and support.

Both the 2000 and 2007 surveys showed that, compared to non-violent women, those who self-reported violence also reported significantly more mental health problems (see Table 3). In the 2000 sample, an increase in the co-morbidity of CMD, from zero to up to six, was associated with increase in the proportion of women reporting violence (see Table 4). However, none of the six CMD conditions, and mental health variables such as depression or PD was included in the set of 8 risk predictors (Table 6). This was probably because the underlying variance captured by traditional mental health variables such as depression, could be captured by variables such as self-harm, and PD by conduct disorder before age 15 and so forth as evidenced by the fact that simple correlation analysis in both 2000 and 2007 samples demonstrated significant positive associations between the CMD variables and each of the eight identified risk factors. Although the direct or indirect relationships between female violence, mental health, and other identified risk factors requires further study, the risk factors identified in the present study can be considered proxy indicators of mental health variables. The advantages of using these proxy indicators in the proposed risk assessment tools is that the ratings do not require specialized training in the assessment of mental disorders and, as such, can be carried out by frontline workers without mental health training. Further study should focus on validating a risk tool using those risk factors and on developing a management and intervention package for women who suffer from mental disorders to ensure referral to appropriate mental health services.

## Conclusions

The prevalence of female violence in the general household population in the UK was low and stable over the 9 year period covered by the surveys. About 80% of violent acts by community women were against their partners or acquaintance, mostly took place at home,in the street or in drinking establishments, and 5% towards children. The eight significant risk factors that can be used to identify violent prone women in the community overlap with risk factors for violent offending and with gender specific risk factors among female offenders. The risk factors are also strong proxy indicators of the presence of significant mental health concerns such as probable affective, substance use and ASPD. The identified risk factors could be aggregated to develop a rating tool that can be easily and rapidly administered by community frontline workers without professional mental health training to identify women at risk for violence and mental health problems. These women could be suitable referrals for mental health and social support and for appropriate interventions to reduce potential harm to themselves and to other, in particular, to family members and to children in their care.

## Endnotes

^a^Ethical approvals for the 2000 survey were obtained from the London Multi-Centre Research Ethics Committee and all local research ethics committees as required [17]. Ethical approval for 2007 survey was obtained from the Royal Free Hospital and Medical School Research Ethics Committee [18].

^b^The list of 52 variables is presented as a supplement material, the Additional file 1.

## Competing interests

The authors declare that they have no competing interests.

## Authors’ contributions

MY designed the study, performed the statistical analysis, interpreted the findings and drafted as well as revised the manuscript. SW assisted in interpreting the findings, reviewed and revised the manuscript. JC contributed to the design of the surveys, assisted in drafting the manuscript. All authors read and approved the final manuscript.

## Pre-publication history

The pre-publication history for this paper can be accessed here:

http://www.biomedcentral.com/1471-2458/13/1020/prepub

## Supplementary Material

Additional file 1 List of variables examined for their association with violence behaviour among women in general households. Click here for file
