# Supplementary material for: Violence, mental health and violence risk factors among women in the general population: an0020epidemiology study based on two national household surveys in the UK
Source: BMC Public Health. 2013 Oct 29;13:1020. doi: 10.1186/1471-2458-13-1020 (PMC3829658; doi:10.1186/1471-2458-13-1020)
Supplement: Additional file 1 — List of variables examined for their association with violence behaviour among women in general households. [file 1471-2458-13-1020-S1.docx]

List of variables examined for their association with violence behaviour among women in general households

| **Variable category** | **Variables** | **Coding** |
| --- | --- | --- |
| Demographics | Ethnicity | White = 1, others =0 |
|  | Education | No qualification =1, any qualification =0 |
|  | Unstable relationship or unmarried | Yes= 1, no = 0 |
|  | Ever had a paid job | Unemployed = 1, others = 0 |
|  | Young age (<30 years) | Yes=1, no=o |
|  | Social class | Social classes I and II = 1, others = 0 |
| Accommodation | Living in Council housing or ever home less | Yes = 1, no=0 |
|  | Area of living | Urban = 1, rural = 0 |
| Well being | Social function | Continuous score |
|  | Nart score for IQ test | Continuous score |
|  | SF12 for general health | Continuous score |
| Axis I disorders | Alcohol dependent | Yes = 1, no = 0 |
|  | Neurotic disorder | Yes = 1, no = 0 |
|  | Any drug dependent | Yes = 1, no = 0 |
|  | Hazardous drinking | Yes = 1, no = 1 |
| Suicide | Ever attempted self-harm/suicide | Yes = 1, no = 0 |
| Axis II disorders (lay screen) | Adult antisocial life style | Yes = 1, no = 0 |
|  | Conduct problems before the age of 15 | Yes = 1 , no = 0 |
|  | Dependent personality disorder | Yes = 1, no = 0 |
|  | Narcissistic personality disorder | Yes = 1, no = 0 |
|  | Histrionic personality disorder | Yes = 1, no = 0 |
|  | Schizoid personality disorder | Yes = 1, no = 1 |
|  | Schizotypal | Yes = 1, no = 0 |
|  | Paranoid personality disorder | Yes = 1, no = 0 |
|  | Obsessive – compulsive personality disorder | Yes = 1 , no = 0 |
|  | Avoidance personality disorder | Yes = 1, no = 0 |
|  | Borderline personality disorder | Yes = 1, no = 0 |
| Childhood adversity / victimization /traumatize | Ever experienced sex abuse | Yes = 1, no = 0 |
|  | Ever experienced domestic violence | Yes = 1, no = 1 |
|  | Bully experience | Yes = 1, no = 0 |
|  | Ever experienced violence at work place | Yes = 1, no = 0 |
|  | Ever homeless | Yes = 1 , no = 0 |
|  | Ever expelled from school | Yes = 1, no = 0 |
|  | Ever ran away from home | Yes = 1, no = 0 |
|  | Institutional care before age of 15 | Yes = 1, no = 1 |
|  | Had finance crisis | Yes = 1, no = 0 |
|  | Had problems with police | Yes = 1, no = 0 |
|  | Traumatised from separation/devoice | Yes = 1 , no = 0 |
|  | Had problematic friends/relatives | Yes = 1, no = 0 |
|  | Illness/assault | Yes = 1, no = 0 |
| Help seeks in last 12 months | GP consultation for psychological problems | Yes = 1, no = 0 |
|  | Seen psychiatrist | Yes = 1 , no = 0 |
|  | Community mental health care service used | Yes = 1, no = 0 |
|  | Seen community psychiatric nurse | Yes = 1, no = 0 |
|  | Counselling treatment for behavior problems | Yes = 1, no = 1 |
|  | Ever psychiatric admission | Yes = 1, no = 0 |
|  | Seen GP for nervous/emotional problem | Yes = 1, no = 0 |
|  | Self-help/support group | Yes = 1 , no = 0 |
|  | Outreach worker | Yes = 1, no = 0 |
|  | Seen psychologist | Yes = 1, no = 0 |
|  | Seen social workers | Yes = 1 , no = 0 |
|  | Long standing illness | Yes = 1, no = 0 |
